# Bacteria-Derived Carbon Dots Inhibit Biofilm Formation of *Escherichia coli* without Affecting Cell Growth

**DOI:** 10.3389/fmicb.2018.00259

**Published:** 2018-02-16

**Authors:** Fengming Lin, Chengcheng Li, Zhan Chen

**Affiliations:** State Key Laboratory of Bioelectronics, School of Biological Science and Medical Engineering, Southeast University, Nanjing, China

**Keywords:** biofilm, carbon dots, lactic acid bacterium, biocompatibility, antifouling

## Abstract

Biofilms are deleterious in many biomedical and industrial applications and prevention of their formation has been a pressing challenge. Here, carbon dots, CDs-LP that were easily synthesized from the biomass of *Lactobacillus plantarum by* one-step hydrothermal carbonization, were demonstrated to prevent biofilm formation of *E. coli*. CDs-LP did not thwart the growth of *E. coli*, indicating the anti-biofilm effect was not due to the bactericidal effect. Moreover, CDs-LP did not affect the growth of the animal cell AT II, showing low cytotoxicity, good safety and excellent biocompatibility. Therefore, CDs-LP could overcome the cytotoxicity issue found in many current antibiofilm agents. CDs-LP represent a new type of anti-biofilm materials, opening up a novel avenue to the development of biofilm treatment.

## Introduction

Biofilms are highly structured communities of immobile microorganisms that are adhered to a surface or to each other, and embedded in a protective extracellular polymeric matrix consisting of polysaccharides, nucleic acids, proteins, and lipids (Flemming et al., 2016). Biofilms are frequently found on natural, clinical and industrial settings, causing tremendous issues due to the fact that they are resistant to external stresses, host defenses and conventional antimicrobial agents (de la Fuente-Núñez et al., 2013). For example, biofilms represent major virulence factors, resulting in chronic human infections, implant failure and even death (Anderson et al., 2003; Duncan et al., 2015). Also, biofilms are harmful for industrial facilities by blocking filtration membranes, fouling marine surfaces, or corroding pipes (Natalio et al., 2012). However, combating these biofilms, by either prevention or eradication, remains particularly challenging in both industrial and biomedical applications.

Annihilation of biofilm *in vivo* with traditional therapeutic antibiotics is becoming an overwhelming task. Antimicrobial therapy alone is rarely effective, and surgical intervention is often needed, resulting in a prolonged course of medical treatment with high costs (Kostakioti et al., 2013). Even worse, the development of new antibiotics is slow and difficult, which cannot keep pace with the emergence of antibiotic-resistant microorganisms (Fischbach and Walsh, 2009). Thus, alternative strategies to combat biofilm are highly desired. With the rapid development of nanotechnology, an increasing number of nanomaterials with unique properties have been explored for fighting various biofilms. Such nanomaterials include metal/metal oxide nanoparticles (Taglietti et al., 2014; Boda et al., 2015; Nguyen et al., 2015; Geilich et al., 2017; Hu et al., 2017) along with polymeric nanoparticles (Duong et al., 2014; Nguyen et al., 2016), nanoparticle-stabilized capsules (Duncan et al., 2015), nanoenzymes (Natalio et al., 2012; Chen et al., 2016), hydrogels(Gao et al., 2014), liposomes (Robinson et al., 2001) and so on, in either antibiotic-free or antibiotic-coated ways. A big concern with the application of nanomaterials in killing biofilm is cytotoxicity which was also often observed in many conventional antibiofilm materials. Despite their excellent anti-biofilm activity, nanomaterials are usually toxic toward microorganisms and human cells, and are consequently not biocompatible (Hu et al., 2017). This non-biocompatibility issue caused safety concerns, decreased therapeutic selectivity, and adverse effects on ecosystems, and thus needs to address urgently. To solve this problem, surface engineering is routinely explored to finely tune nanomaterials' surface properties to achieve concurrent antibiofilm activity and non-cytotoxicity, which nevertheless involves the use of complex chemical/physical modification methods (Giri et al., 2015; Hu et al., 2017).

**Scheme 1:**
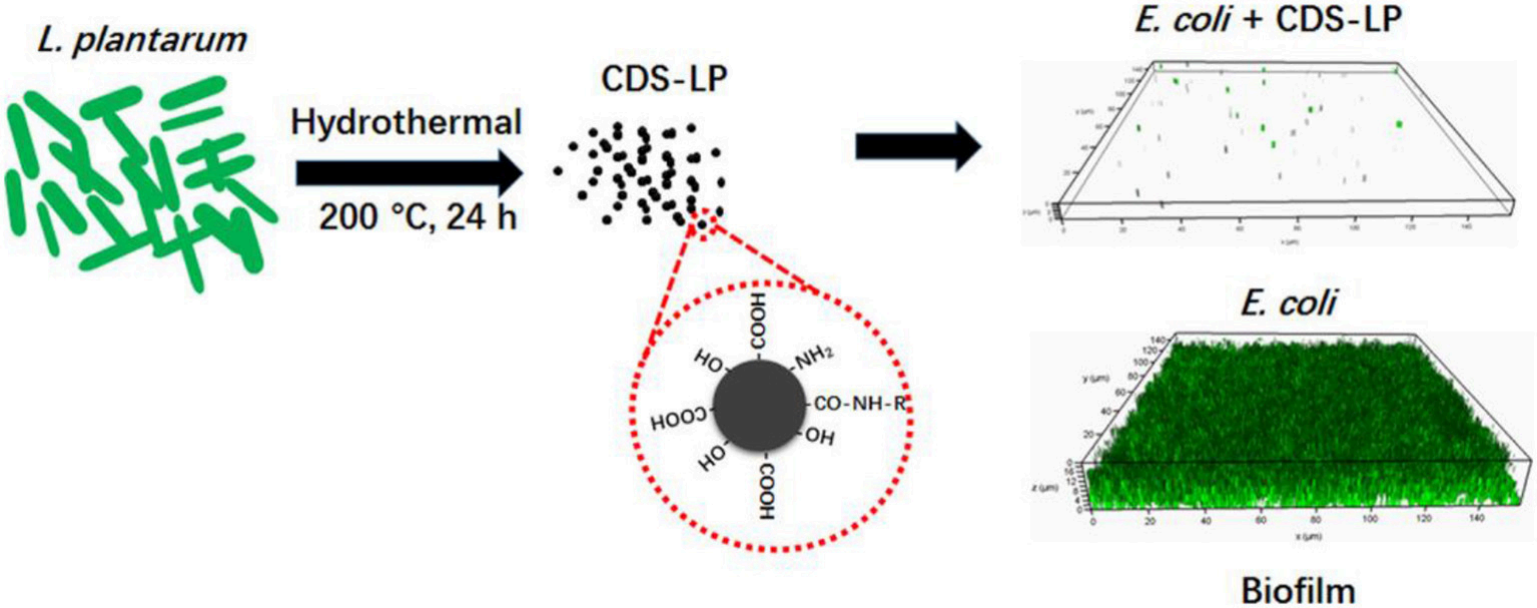
Schematic of the synthetic route and anti-biofilm of CDs-LP.

In this study, we present a novel type of carbon dots, easily prepared using *L. plantarum* as a single carbon source by one-step hydrothermal reaction without any chemical/physical modification, to prevent *E. coli* biofilm formation with excellent biocompatibility (Scheme 1). Carbon dots have wide applications in many fields including sensing, bio-imaging, photo-catalysis, drug delivery, etc., because of their facile synthesis route, exceptional photostability, low toxicity, great water dispersibility and amenable surface modification (Li et al., 2012; Wang and Hu, 2014). They have been successfully employed to discriminate gram negative/positive bacteria (Yang et al., 2016) as well as live/dead microorganisms (Hua et al., 2017b). The amphiphilic carbon dots have been synthesized and utilized for efficiently labeling biomimetic and cellular membranes (Nandi et al., 2014, 2015). Furthermore, carbon dots usually possess varied surface functional groups and can be easily engineered by other drugs and ligands for the applications in organelle-targeted imaging and/or drug delivery (Yang et al., 2015; Zhang et al., 2015; Hua et al., 2017a). For biofilm application, carbon dots have been successfully exploited to image biofilm matrix (Ritenberg et al., 2016) and biofilm-encased microorganisms (Lin et al., 2017). However, to the best of our knowledge, the utilization of carbon dots in combating biofilm has not been reported.

## Materials and methods

### Preparation and purification of CDs

CDs-LP were made from *L. plantarum* LLC-605 by one-step hydrothermal carbonization. *L. plantarum* LLC-605 was isolated in our lab from the traditional Chinese fermented food FuYuan pickles (Yunnan, China), which was deposited in NCBI with the accession number KX443590 (Li et al., 2017). *L. plantarum* LLC-605 was cultured in 30 mL De Man, Rogosa and Sharpe (MRS) medium at 31°C for 18 h without shaking. The overnight cell culture was centrifuged at 5,000 rpm for 10 min to remove the supernatant, washed three times with Milli-Q water, and transferred into a 50 mL Teflon-lined stainless-steel autoclave, and re-suspended in 30 mL Milli-Q water. The autoclave was incubated at 200°C for 24 h. To remove large particles/aggregates, the synthesized dark brown solution was centrifuged at 12,000 rpm for 10 min, and the supernatant was filtered through a 0.22 μm filter membrane. Then, the CD solution was dialyzed against Milli-Q water in a dialysis bag with molecular weight cut-off of 1 kDa for 2 days, and stored at 4°C for further experiments. For quantification and long-term storage, the CDs were lyophilized and weighed after dialysis.

### Characterizations of CDs

The morphology, size and element components of the as-synthesized CDs-LP were determined. A drop of CDs-LP solution was deposited on a 400-mesh carbon-coated copper grid and examined by a transmission electron microscope (TEM, JEM-2100, JEOL Ltd., Japan). Ultraviolet–visible (UV–vis) and fluorescence spectra of CDs-LP in pure water or phosphate-buffered saline (PBS: 137 mM NaCl, 2.7 mM KCl, 10.1 mM Na_2_HPO_4_, 1.7 mM KH_2_PO_4_, pH 7.4) were obtained by a UV–vis spectrophotometer (UV-2600, Shimadzu, Japan) and a spectrofluorophotometer (RF-5301PC, Shimadzu, Japan), respectively. Fourier transform infrared (FTIR) spectroscopic experiment was carried out with an FTIR spectrometer (Nicolet iS50, Thermo Scientific, USA). X-ray photoelectron spectroscopic (XPS) experiment was performed via a Japan Kratos Axis Ultra HAS spectrometer. Zeta potential values of CDs-LP were determined with a Zetasizer instrument (Malvern Instruments, Nano ZS, United Kingdom). The fluorescence quantum yield (QY) of the CDs-LP was calculated according to the method previously reported (Yang et al., 2015).

### Inhibition of *E. coli* biofilm formation

Bacteria were cultured overnight in LB at 37°C with 180 rpm. The overnight bacterial cell culture was diluted 1:100 in 1/5 LB without and with different concentrations of CDs-LP and then inoculated into the 96-well plates (Costar, Corning, USA) with 100 uL/well or the glass bottom cell culture dish (NEST, USA) with 2 mL/dish. The inoculated 96-well plates and dishes were incubated for 1–5 days at 28°C without shaking (Jurcisek et al., 2011; Duncan et al., 2015).

### Confocal imaging

For confocal imaging, static biofilm was cultured and processed inside glass bottom culture dishes (Nest, USA) following the methods as described above. The grown biofilms were washed with PBS three times, stained with a live/dead assay (Filmtracer LIVE/DEAD Biofilm Viability Kit, Thermo Fisher Scientific) and then examined under a confocal microscope (TCS SP8, Leica, Germany). Fluorescence images were acquired with excitation at 488 nm, and the corresponding emissions were detected at 500 and 635 nm, respectively.

### Crystal violet assay

Static biofilm formed and processed as described above was carefully washed three times with PBS (Jurcisek et al., 2011; Duncan et al., 2015). The biofilm was heat-fixed at 60°C for 1 h, stained with crystal violet for 10 min, and then rinsed with Milli-Q water until rinse water was clear. After removing residual fluid, the stained biofilm was photographed or solubilized in the destaining solution (10% methanol and 7.5% acetic acid in water). The absorbance at 595 nm was read on a microplate reader (Multiskan, Thermo Scientific). The experiments were carried out with eight biological replicates.

### Effect of CDs-LP on *E. coli* growth

Bacteria were cultured overnight in LB at 37°C with 180 rpm. The overnight bacterial cell culture was diluted 1:100 in 1/5 lysogeny broth (LB) without and with different concentrations of CDs-LP and then inoculated into the 96-well plates (Costar, Corning, USA) with 100 uL/well. The inoculated 96-well plates and dishes were incubated for 24 h at 28°C without shaking. Before measuring the optical density (OD) at 600 nm, the cultured bacteria were well resuspended by pipetting up and down, and then the OD at 600 nm was recorded.

In addition, colony forming unit (CFU) counting method (plate count method) was utilized to evaluate the cytotoxicity of CDs-LP to *E. coli*. Briefly, *E. coli* in log phase were cultured in 1/5 LB containing 6 mg/mL CDs-LP at 28°C for 24 h. Each culture was diluted with 1/5 LB with a dilution factor of 1 × 10^5^. Then the diluted microbial cells were plated in triplicate on LB agar plates and cultured at 37°C for 24 h. Finally, the cell colonies of *E. coli* were counted. Three biological replicates were performed. The cytotoxicity was evaluated by comparing the number of CFUs in treated groups to that in the control group.

### MTT assay

AT II cells (normal lung cells) were cultured at 37°C with 5% CO_2_ in DMEM cell media supplemented with 10% fetal bovine serum. The cells (5 × 10^4^ cells per well) were seeded into a 96-well plate, cultured overnight and then treated with different concentrations of CDs-LP (0, 0.01, 0.05, 0.1, 0.5, 1, 3, and 6 mg/mL) for 24 h. 10 μL 3-(4,5-dimethylthiazol-2-yl)-2,5-diphenyltetrazolium bromide (MTT, 5 mg/mL) was added to each well. After incubation for 4 h, the culture medium in each well was replaced with 150 μL dimethyl sulfoxide (DMSO). The absorbance at 492 nm was recorded by a Multiskan FC microplate photometer (Thermo).

## Results and discussion

### Preparation and characterization of carbon dots

**Figure 1:**
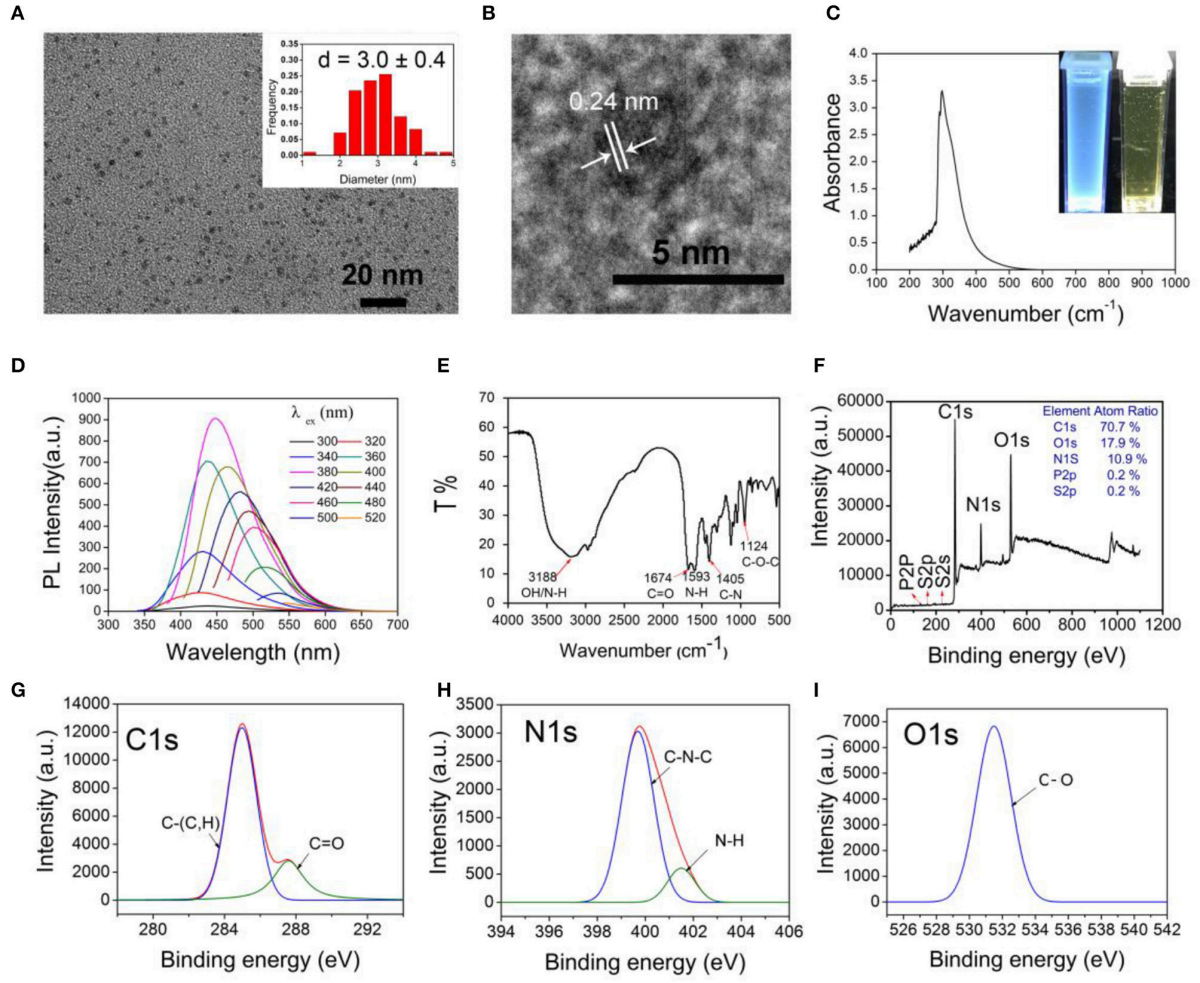
Characterization of CDs-LP. **(A)** TEM image and the corresponding size distribution histogram of CDs-LP (inset). **(B)** The high-resolution TEM image of CDs-LP. **(C)** UV–vis absorption spectra of CDs-LP in water. The insets are photographs of CDs-LP in water with and without irradiation under a 365 nm UV lamp. **(D)** Fluorescence spectra of CDs-LP dispersed in water. **(E)** FTIR spectra of dried CDs-LP. **(F)** XPS spectrum of dried CDs-LP and the high-resolution XPS peaks of C1s **(G)**, N1s **(H)** and O1s **(I)**.

*L. plantarum* LLC-605 is a highly exopolysaccharide-producing strain (Li et al., 2017), and might contain a great number of carbohydrates, which can serve as abundant precursors for carbon dots formation together with other biological molecules like proteins and peptides in the bacterium. Furthermore, it belongs to lactic acid bacteria that have healthy benefits for humans. Therefore, carbon dots were fabricated using *L. plantarum* LLC-605 via one-step hydrothermal carbonization, which is denoted as CDs-LP. CDs-LP were quasi-spherical with an average diameter of 3 nm as demonstrated in the TEM image (Figure 1A). A crystalline structure can be observed in the high-resolution image of CDs-LP with the lattice fringe of 0.32 nm, in consistent with the (002) spacing reported for graphitic carbon (Reckmeier et al., 2016) (Figure 1B). CDs-LP could be homogeneously dispersed in aqueous solution and emitted blue fluorescence under 365 nm UV light irradiation (Figure 1C). Besides, no characteristic absorption peak was found in the UV-vis spectrum of CDs-LP, which was also observed in other carbon nanomaterials (Zhao et al., 2011; Jiang et al., 2017; Lin et al., 2017). The synthesis mechanism of CDs remains obscure, which might involve decomposing and carbonization of stocks, dehydration condensation, nucleation and growth of carbon in the progress of hydrothermal experiments (Reckmeier et al., 2016).

The quantum yield (QY) of CDs-LP was 10.3%. CDs-LP has a largely negative charge with a zeta potential of −22 mV, which might due to that its raw material L. plantarum LLC-605 is negatively charged. The emission intensity of CDs-LP increased gradually from 300 nm to 380 nm, and then declined from 400 to 520 nm (Figure 1D). Meanwhile, the emission peaks of CDs-LP gradually red-shifted from 420 to 540 nm when the excitation wavelength varied from 300 to 520 nm (Figure 1D). The maximum emission peak was at 450 nm with the excitation wavelength of 380 nm. Obviously, the fluorescence emission spectrum of CDs-LP is dependent on the excitation wavelength. Although the mechanism behind the red-shift emission property that harbored by a great number of the reported carbon dots (Wang and Zhou, 2014; Yang et al., 2016; Hua et al., 2017b) is still not clear, it is often associated with surface- or defect states in the amorphous carbon shell of CDs near the Fermi level (Reckmeier et al., 2016; Hua et al., 2017b).

FITR and XPS were utilized to investigate the composition of CDs-LP. As shown in Figure 1E, the broad absorption band around 3,188 cm^−1^ was due to the stretching vibrations of O–H (ν(O–H)) and/or N–H (ν(N–H)). The peak at 1,674 cm^−1^ belongs to the C = O stretching vibration, ν(C = O), which may come from the amide bond (–CONH–) or the –COOH group. The peak at 1,593 cm^−1^ is attributable to the N–H bending vibration δ(N–H), owing to the amide bond (–CONH–) or the NH_2_ group. The peak at 1,405 cm^−1^ may derive from the C–H bending vibration (δ(C–H)), and the peak at 1,124 cm^−1^ can be assigned to the C–O stretching vibration (ν(C–O)). CDs-LP contain the elements of C (70.7%), O (17.9%), N (10.9%), P (0.2%), and S (0.2%) as measured by XPS (Figure 1F). Note that the H element cannot be determined by XPS. The two fitted peaks at 285 and 287.6 eV in the high-resolution C1s spectrum (Figure 1G) can be assigned to carbon-containing groups C–C/C = C (sp2 carbons) and C = O, respectively (Bao et al., 2015). The N1s peak at 399.7 and 401.5 eV (Figure 1H) could separately be ascribed to amide nitrogen C-N-C and the amino nitrogen N-H (Yang et al., 2014; Zhang and Chen, 2014), evidencing the presence of both amino and amido groups in CDs-LP as found by FTIR. For the O1s spectrum, the fitted peak at 531.5 eV (Figure 1I) is attributed to the oxygen element in the form of C–O (Zhang and Chen, 2014; Ding et al., 2016). In conclusion, the FTIR and XPS results showed that the as-synthesized CDs-LP contain the elements C, H, O, N, P, and S, and the functional groups –OH, –COOH, –NH_2_, and –CONH–.

### CDs-LP inhibit the formation of *E. coli* biofilm

**Figure 2:**
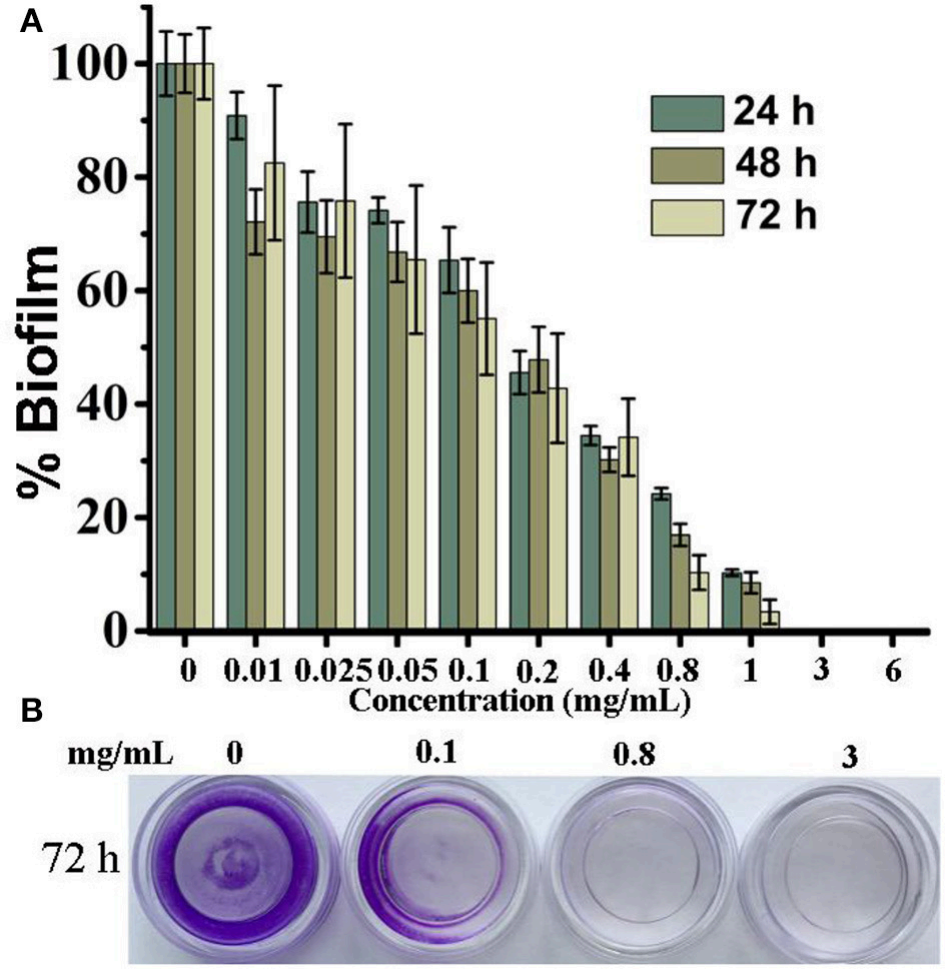
The effect of CDs-LP on the *E. coli* biofilm was assayed by the crystal violet method. The *E. coli* biofilm was grown at 28°C in 1/5 LB with the treatment of different concentrations of CDs-LP for different times as indicated in the figure. The absorbance of *E. coli* biofilm in the absence of CDs-LP at 24, 48, and 72 h after staining by the crystal violet, was recorded at 595 nm and arbitrarily assigned as 100%, respectively **(A)**. Also, the *E. coli* biofilms with the treatment of 0, 0.1, 0.8, or 3 mg/mL CDs-LP for 72 h were stained by the crystal violet and photographed **(B)**.

**Figure 3:**
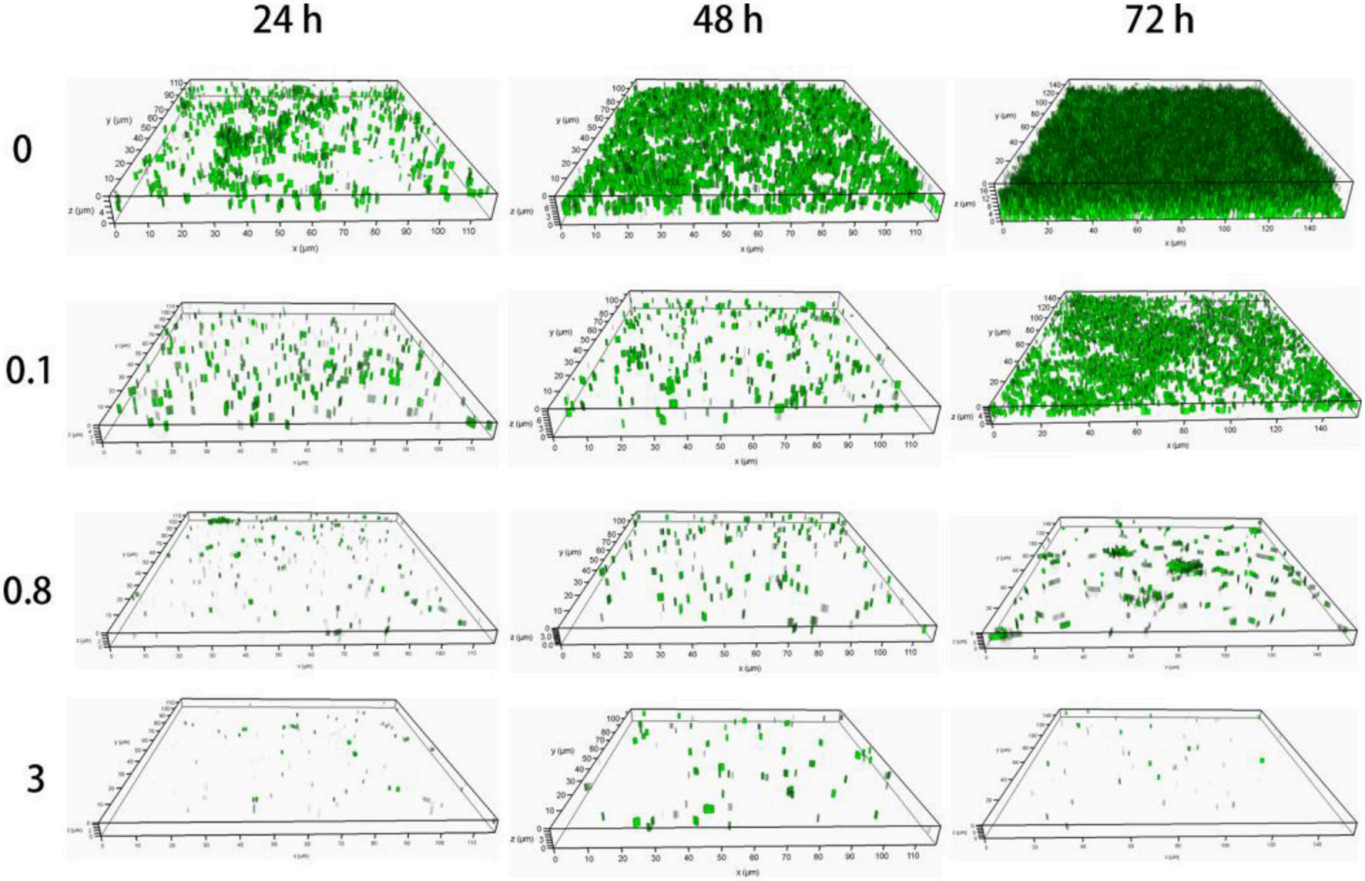
The 3D images of *E. coli* biofilms at 24, 48, and 72 h in the presence of 0, 0.1, 0.8, or 3 mg/mL CDs-LP were observed under a confocal laser microscope. The biofilms were stained using the live/dead stain assay. Green and red dots represent live and dead bacteria, respectively.

To observe how CDs-LP affect the formation of *E. coli* biofilms, various amounts of carbon dots were added into the culture media during biofilm growth and the processes of biofilm formation within 72 h were monitored by the crystal violet assay (Figure 2). Before being assayed by the crystal violet assay (Figure 2) or checked under confocal microscopy (Figure 3), the samples were washed three times with PBS to remove the planktonic cells. So if the biofilm formation is inhibited and the cells do not attach to the surface, the cells would be washed away, leaving fewer cells on the surface. The biofilm formation was reduced gradually from 100% to 10.2% in 24 h, as the CDs-LP concentration increased from 0 to 1 mg/mL (Figure 2A). When the concentration of CDs-LP was 3 mg/mL and higher, no *E. coli* biofilm was formed, showing that CDs-LP could effectively inhibit the biofilm formation of *E. coli* when the concentration is above 3 mg/mL. When the treatment time was increased from 24 to 72 h, the inhibition effect was enhanced slightly (Figure 2A). The mean IC50 value for biofilm inhibition (MBIC50) is defined as the lowest concentration at which at least 50% reduction in biofilm formation was achieved compared to untreated biofilm (Feldman et al., 2012). Here the MBIC50 of CDs-LP was measured to be 0.4 mg/mL. Moreover, the crystal violet staining results of the *E. coli* biofilms with the treatment of 0, 0.1, 0.8, and 3 mg/mL CDs-LP for 72 h are shown in Figure 2B. Clearly, the presence of CDs-LP significantly reduced the biofilm formation of *E. coli* at 0.1 mg/ mL and completely inhibited it at 0.8 or 3 mg/mL, which was in line with the results obtained by measuring the absorbance at 595 nm (Figure 2A).

Although carbon dots have been successfully exploited in imaging biofilm matrix (Ritenberg et al., 2016) and biofilm-encased microorganisms (Lin et al., 2017), it is for the first time to report that carbon dots can inhibit *E. coli* biofilm formation. Moreover, the synthesis of CDs-LP only requires *L. plantarum* LLC-605 as the only raw material through one-step hydrothermal reaction, which is very facile and renewable. *L. plantarum* LLC-605 belongs to lactic acid bacteria that have healthy benefits for humans. In biomedical practice, the successful prevention of bacterial biofilm formation could reduce the need for interventional treatments such as systemic and local antibiotic administration, amputation, debridement, or reconstruction, which would improve patient outcomes and reduce the costs associated with infection treatment. In industrial applications, antifouling is important for the performance and durability of the facilities, and cost saving. Therefore, the development of new biofilm inhibitors like CDs-LP here is of great value.

### CDs-LP did not inhibit the growth of *E. coli*

Confocal microscopy was utilized to evaluate the ability of CDs-LP to prevent adhesion and colonization of *E. coli*. The live and dead bacteria were stained with green and red respectively with the live/dead stain assay (Figure 3). After treatment with CDs-LP of 0.1 mg/mL, 0.8 mg/mL, or 3 mg/mL, the *E. coli* biofilm became thinner with a significant reduction in biovolume, as compared to the untreated control. Though several *E. coli* cells were observed on the culture dish's surface at 0.8 or 3 mg/mL CDs-LP concentration, they are not able to colonize and aggregate to form biofilm. Interestingly, no red fluorescence was observed (Figure 3), showing that there was no dead *E. coli* cells inside the biofilm and indicating CDs-LP caused no toxicity to *E. coli* cells. Seemingly, the presence of CDs-LP prevents the colonization and aggregation of *E. coli* on the surface that are prerequisites for *E. coli* biofilm formation while not killing the cells.

**Figure 4:**
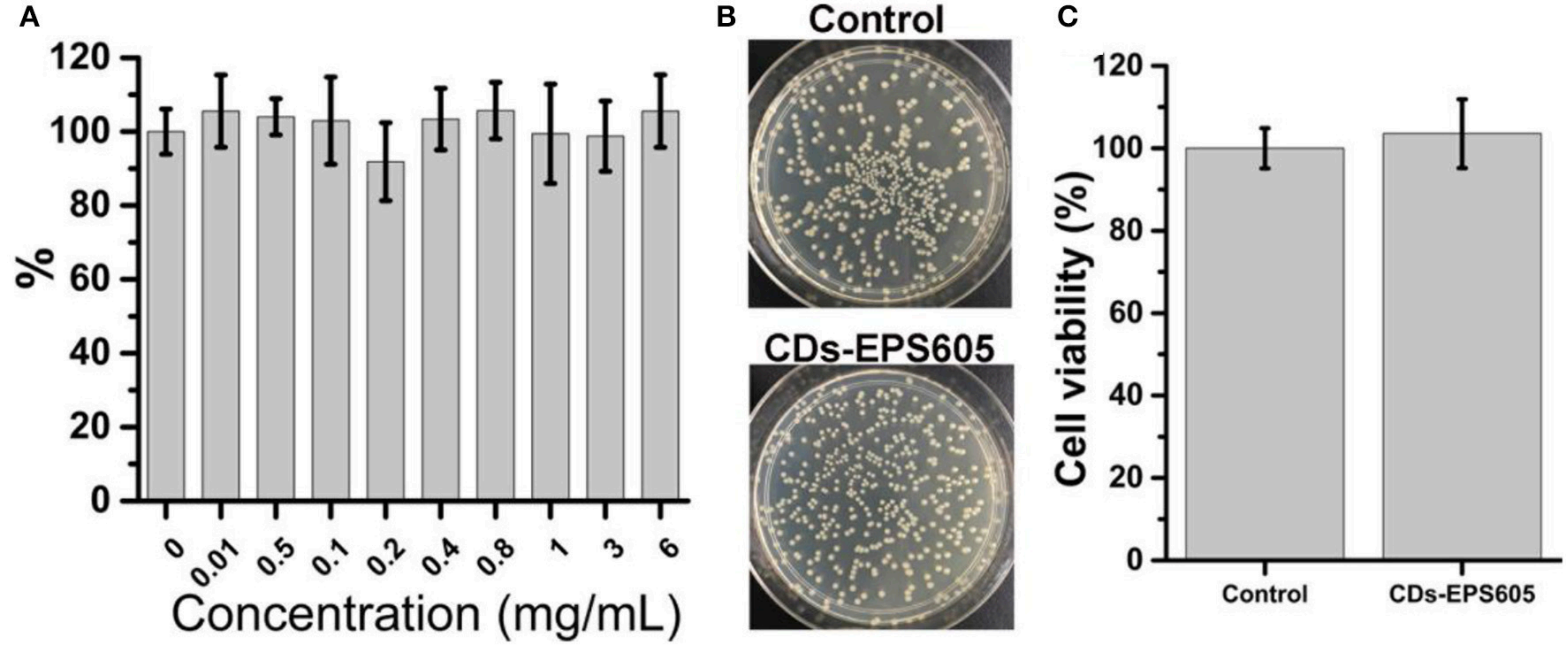
Effect of CDs-LP on *E. coli* growth. **(A)** Growth of *E. coli* for 24 h in 1/5 LB containing 0-6 mg/mL CDs-LP at 28°C without shaking. The absorbance of *E. coli* cell culture in the absence of CDs-LP was measured at 600 nm and arbitrarily defined as 100%. **(B)** Photographs of the agar plates and **(C)** corresponding statistical histograms of colonies of *E. coli* cultured in 1/5 LB (control) and CDs-LP (6 mg/mL) for 24 h.

To further confirm that the capability of CDs-LP to inhibit biofilm formation is not due to bactericidal activity, the effect of CDs-LP on the growth of *E. coli* was tested by both optical density assay and plate count method (Figure 4). The OD of the *E. coli* culture remained unaltered in the presence of 0–6 mg/mL CDs-LP (Figure 4A). Moreover, the cell viability of *E. coli* incubated with 6 mg/mL CDs-LP for 24 h were about 103% (Figures 4B,C). These results demonstrate that CDs-LP did not block the *E. coli* growth, which is well correlated with the data obtained by the live/dead assay (Figure 3). Obviously, the anti-*E. coli* biofilm property of CDs-LP was not attributed to a bactericidal effect, which would be beneficial for the growth of natural bacterial flora. Identification of anti-biofilm compounds that prevent biofilm formation without killing bacteria will provide a much flexible and practical solution to biofilm infections, because such an approach will not interfere the other non-infectious and natural bacterial flora which are crucial for human and ecosystems. Meanwhile, antifouling coatings nontoxic to marine biota, such as silicone elastomers (Phanindhar et al., 2015) and vanadium pentoxide nanowires (Natalio et al., 2012), have been of great interest, for they do not cause deleterious effects in aquatic environments and are environmentally benign (Lejars et al., 2012). In addition, the non-bactericidal property of CDs-LP implies that the application of CDs-LP in anti-biofilm should not lead to antibiotic resistance that long haunts antibiotic-based antibiofilm agents. When it is necessary to kill microorganisms, CDs-LP can be simultaneously used in combination with antibiotics. Therefore, preventing *E. coli* biofilm formation by CDs-LP represents a neutral and flexible strategy.

### The CDs-LP is non-toxic toward animal cells

**Figure 5:**
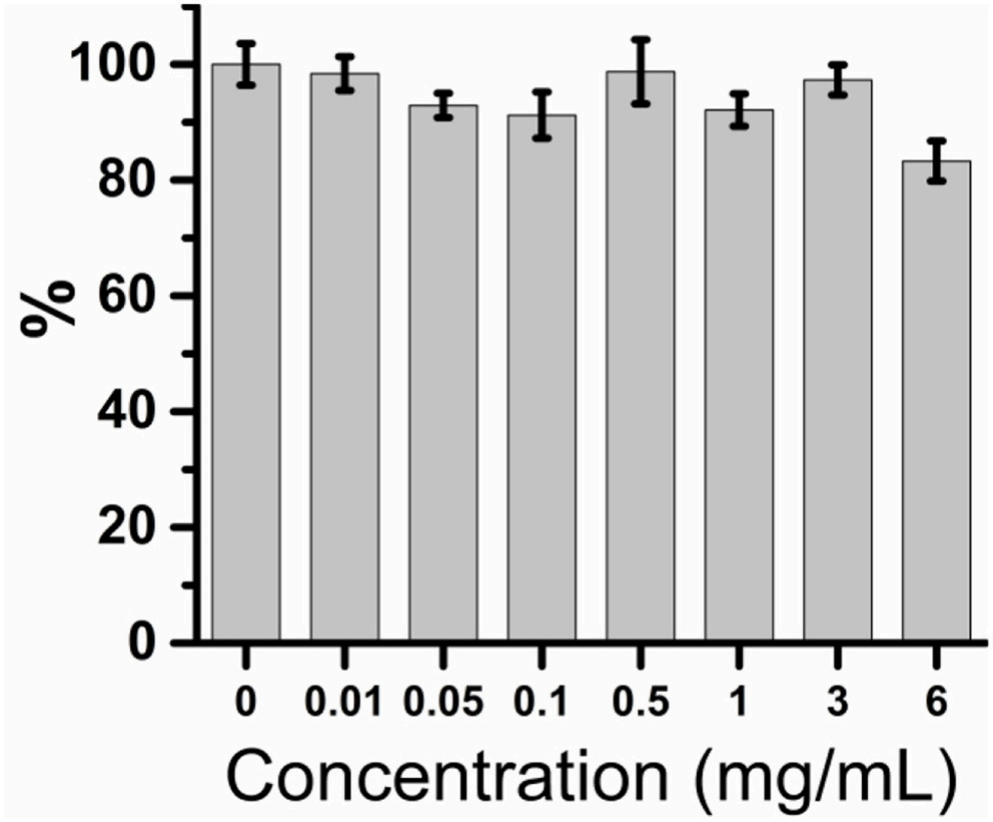
*In vitro* safety testing result of CDs-LP. Relative viabilities of AT II cell exposed to different concentrations of CDs-LP were determined by MTT assay. The absorbance of AT II cells in the absence of CDs-LP was measured at 492 nm and arbitrarily assigned as 100%.

To evaluate the safety of CDs-LP, which is important for both biomedical and industrial applications, the relative viabilities of AT II cell with the treatment of CDs-LP in the range from 0 to 6 mg/mL were measured (Figure 5). More than 80% of AT II cells remained alive under all the tested concentrations of CDs-LP in the range of 0–6 mg/mL, implying that CDs-LP is non-toxic within the concentrations that are utilized to inhibit *E. coli* biofilm formation. The formation of *E. coli* biofilm was inhibited completely by CDs-LP at 1 mg/mL and higher concentrations. Given that CDs-LP are negatively charged and CDs are well known for their excellent biocompatibility, it is reasonable that CDs-LP did not affect the growth and viability of neither bacteria nor the animal cells.

Several biofilm inhibitors have been reported to be non-toxic to *E. coli* and other bacteria as well as animal cells, such as (5Z)-4-bromo-5-(bromomethylene)-3-butyl-2(5H)-furanone (furanone) (Ren et al., 2001), ursolic acid (Ren et al., 2005), 5-fluorouracil(Attila et al., 2009), indole derivatives (Lee et al., 2007, 2011, 2012) and cationic pillararenes (Joseph et al., 2016). They inhibit the biofilm formation through cell signaling, sulfur metabolism (Wood, 2009) or cysteine metabolism (Lee et al., 2007). Most of these biofilm inhibitors are natural compounds that are not easy to obtain due to scarce resources, while others are synthesized with complicated chemical/physical methods that are not easy to scale up. Contrarily, here the synthesis of CDs-LP is simple, fast and in large amount using the raw material strain LCC605 that is easily accessible. Furthermore, there are active functional groups on CDs-LP surfaces as detected by FTIR and XPS, allowing for further surface engineering to achieve desirable surface coating and bactericidal property.

Most of the current anti-biofilm nanomaterials exhibit potential cytotoxicity and poor biocompatibility, which is mainly caused by the positive charge of cationic nanomaterials that can interact with the negatively charged bacteria/mammalian cell surface through electrostatic interaction. Surface engineering has been often explored to solve this issue. For example, carbon nano/microspheres have been combined with metal nanoparticles to reduce their toxicity and improve their stability (Cui et al., 2012; Li et al., 2014; Cheng et al., 2015). The surface charge of gold nanoparticles was modulated to achieve specific toxicity toward bacterial biofilms while minimizing mammalian cytotoxicity (Giri et al., 2015). Healthy tissues under generally physiological conditions have a pH of ~7.4, whereas biofilm is acidic with a pH of ~5.5. Therefore nanoparticles were modified with pH-responsive moieties that enable specific target of biofilm and/or drug release at acidic pH found within biofilm, leading to highly efficient antibiofilm activity without damaging the tissues around biofilm (Horev et al., 2015; Liu et al., 2016; Hu et al., 2017). Clearly, these surface engineering strategies usually involved complicated chemical/physical modifications. Conversely, the one-step hydrothermal synthesis of CDs-LP reported here is very simple. Given that the most-explored metal-containing nanomaterials for biofilm treatment have potential cytotoxicity and poor biocompatibility, it is of great importance to develop the metal-free, carbon-based materials that are less toxic and more biocompatible, as demonstrated by CDs-LP in our study.

CDs-LP did not show bactericidal activity toward *E. coli*, but it could significantly reduce the colonization and aggregation of *E. coli* on the surface that are essential for *E. coli* biofilm formation. Instead of preventing biofilm formation through disrupting cell membranes and killing bacteria by many nanomaterials which might cause cytotoxicity (Cui et al., 2012; Li et al., 2014; Cheng et al., 2015; Giri et al., 2015; Horev et al., 2015; Liu et al., 2016; Hu et al., 2017), here CDs-LP likely inhibit the biofilm formation through cell signaling (Wood, 2009), sulfur metabolism(Wood, 2009) or cysteine metabolism (Lee et al., 2007), as found for other non-toxic biofilm inhibitors. However, it is not unclear how and which signal pathways in *E. coli* are affected by CDs-LP and involved in the biofilm inhibition. Further detailed study is required but beyond the scope of this research.

## Conclusion

In this study, a new type of carbon dots, CDs-LP, was synthesized by one-step hydrothermal carbonization of *L. plantarum* LLC-605, which is facile, fast, cheap, and green. CDs-LP are capable of inhibiting *E. coli* biofilm formation. Most importantly, CDs-LP did not inhibit the growth of *E. coli* and mammalian cells, preventing the generation of bacteria drug-resistance and presenting great biocompatibility. CDs-LP represent a novel kind of non-toxic anti-biofilm materials to circumvent the cytotoxicity faced by many current anti-biofilm agents, holding great promise for eco-friendly applications against *E. coli* biofilm formation for both microbial infection treatment and anti-biofouling material development.

## Author contributions

FL conceived and designed the study. FL carried out the majority of the experiments. CL conducted the characterization of carbon dots. FL and ZC analyzed the data and drafted the manuscript. All authors read and approved the final manuscript.

### Conflict of interest statement

The authors declare that the research was conducted in the absence of any commercial or financial relationships that could be construed as a potential conflict of interest.

## References

[B1] Anderson G. G. Palermo J. J. Schilling J. D. Roth R. Heuser J. Hultgren S. J. (2003). Intracellular bacterial biofilm-like pods in urinary tract infections. Science 301, 105–107. 10.1126/science.1084550 12843396

[B2] Attila C. Ueda A. Wood T. K. (2009). 5-Fluorouracil reduces biofilm formation in *Escherichia coli* K-12 through global regulator AriR as an antivirulence compound. Appl. Microbiol. Biotechnol. 82, 525–533. 10.1007/s00253-009-1860-8 19172264

[B3] Bao L. Liu C. Zhang Z. L. Pang D. W. (2015). Photoluminescence-tunable carbon nanodots: surface-state energy-gap tuning. Adv. Mater. Weinheim. 27, 1663–1667. 10.1002/adma.201405070 25589141

[B4] Boda S. K. Broda J. Schiefer F. Weber-Heynemann J. Hoss M. Simon U.. (2015). Cytotoxicity of ultrasmall gold nanoparticles on planktonic and biofilm encapsulated Gram-positive Staphylococci. Small 11, 3183. 10.1002/smll.201403014 25712910

[B5] Chen Z. Ji H. Liu C. Bing W. Wang Z. Qu X. (2016). A multinuclear metal complex based dnase-mimetic artificial enzyme: matrix cleavage for combating bacterial biofilms. Angew Chem. Int. Ed. 55, 10732–10736. 10.1002/anie.201605296 27484616

[B6] Cheng X. Fu A. Li H. Wang Y. Guo P. Liu J. (2015). Sustainable preparation of copper particles decorated carbon microspheres and studies on their bactericidal activity and catalytic properties. ACS Sust. Chem. Eng. 3, 2414–2422. 10.1021/acssuschemeng.5b00382

[B7] Cui J. Hu C. Yang Y. Wu Y. Yang L. Wang Y. (2012). Facile fabrication of carbonaceous nanospheres loaded with silver nanoparticles as antibacterial materials. J. Mater. Chem. 22, 8121–8126. 10.1039/c2jm16441h

[B8] de la Fuente-Núñez C. Reffuveille F. Fernandez L. Hancock R. E. (2013). Bacterial biofilm development as a multicellular adaptation: antibiotic resistance and new therapeutic strategies. Curr. Opin. Microbiol. 16, 580–589. 10.1016/j.mib.2013.06.013 23880136

[B9] Ding H. Yu S. B. Wei J. S. Xiong H. M. (2016). Full-color light-emitting carbon dots with a surface-state-controlled luminescence mechanism. ACS Nano 10, 484–491. 10.1021/acsnano.5b05406 26646584

[B10] Duncan B. Li X. Landis R. F. Kim S. T. Gupta A. Wang L. S.. (2015). Nanoparticle-stabilized capsules for the treatment of bacterial biofilms. ACS Nano 9, 7775–7782. 10.1021/acsnano.5b01696 26083534 PMC5047390

[B11] Duong H. T. Jung K. Kutty S. K. Agustina S. Adnan N. N. Basuki J. S.. (2014). Nanoparticle (star polymer) delivery of nitric oxide effectively negates *Pseudomonas aeruginosa* biofilm formation. Biomacromolecules 15, 2583–2589. 10.1021/bm500422v 24915286

[B12] Feldman M. Tanabe S. Howell A. Grenier D. (2012). Cranberry proanthocyanidins inhibit the adherence properties of Candida albicans and cytokine secretion by oral epithelial cells. BMC Complement. Altern. Med. 12:6. 10.1186/1472-6882-12-6 22248145 PMC3273432

[B13] Fischbach M. A. Walsh C. T. (2009). Antibiotics for emerging pathogens. Science 325, 1089. 10.1126/science.1176667 19713519 PMC2802854

[B14] Flemming H. C. Wingender J. Szewzyk U. Steinberg P. Rice S. A. Kjelleberg S. (2016). Biofilms: an emergent form of bacterial life. Nat. Rev. Microbiol. 14, 563–575. 10.1038/nrmicro.2016.94 27510863

[B15] Gao W. Vecchio D. Li J. Zhu J. Zhang Q. Fu V.. (2014). Hydrogel containing nanoparticle-stabilized liposomes for topical antimicrobial delivery. ACS Nano 8, 2900–2907. 10.1021/nn500110a 24483239 PMC4004330

[B16] Geilich B. M. Gelfat I. Sridhar S. van de Ven A. L. Webster T. J. (2017). Superparamagnetic iron oxide-encapsulating polymersome nanocarriers for biofilm eradication. Biomaterials 119, 78–85. 10.1016/j.biomaterials.2016.12.011 28011336

[B17] Giri K. Yepes L. R. Duncan B. Parameswaran P. K. Yan B. Jiang Y.. (2015). Targeting bacterial biofilms via surface engineering of gold nanoparticles. RSC Adv. 5, 105551–105559. 10.1039/C5RA16305F 26877871 PMC4748853

[B18] Horev B. Klein M. I. Hwang G. Li Y. Kim D. Koo H.. (2015). pH-activated nanoparticles for controlled topical delivery of farnesol to disrupt oral biofilm virulence. ACS Nano 9, 2390–2404. 10.1021/nn507170s 25661192 PMC4395463

[B19] Hu D. Li H. Wang B. Ye Z. Lei W. Jia F.. (2017). Surface-adaptive gold nanoparticles with efective adherence and enhanced photothermal ablation of methicillin-resistant *Staphylococcus aureus* biofilm. ACS Nano. 11, 9330–9339. 10.1021/acsnano.7b04731 28806528

[B20] Hua X. W. Bao Y. W. Chen Z. Wu F. G. (2017a). Carbon quantum dots with intrinsic mitochondrial targeting ability for mitochondria-based theranostics. Nanoscale 9, 10948–10960. 10.1039/C7NR03658B 28736787

[B21] Hua X. W. Bao Y. W. Wang H. Y. Chen Z. Wu F. G. (2017b). Bacteria-derived fluorescent carbon dots for microbial live/dead differentiation. Nanoscale, 9, 2150–2161. 10.1039/C6NR06558A 27874123

[B22] Jiang Y. W. Gao G. Zhang X. Jia H. R. Wu F. G. (2017). Antimicrobial carbon nanospheres. Nanoscale 9, 15786–15795. 10.1039/C7NR04679K 28819664

[B23] Joseph R. Naugolny A. Feldman M. Herzog I. M. Fridman M. Cohen Y. (2016). Cationic pillararenes potently inhibit biofilm formation without affecting bacterial growth and viability. J. Am. Chem. Soc. 138, 754. 10.1021/jacs.5b11834 26745311

[B24] Jurcisek J. A. Dickson A. C. Bruggeman M. E. Bakaletz L. O. (2011). *In vitro* biofilm formation in an 8-well chamber slide. J. Vis. Exp. 2011:2481 10.3791/2481 PMC3182645 21304464

[B25] Kostakioti M. Hadjifrangiskou M. Hultgren S. J. (2013). Bacterial biofilms: development, dispersal, and therapeutic strategies in the dawn of the postantibiotic era. Cold Spring Harb. Perspect. Med. 3:a010306. 10.1101/cshperspect.a010306 23545571 PMC3683961

[B26] Lee J. H. Cho M. H. Lee J. (2011). 3-indolylacetonitrile decreases *Escherichia coli* O157:H7 biofilm formation and *Pseudomonas aeruginosa* virulence. Environ. Microbiol. 13, 62–73. 10.1111/j.1462-2920.2010.02308.x 20649646

[B27] Lee J. H. Kim Y. G. Kim C. J. Lee J. C. Cho M. H. Lee J. (2012). Indole-3-acetaldehyde from Rhodococcus sp. BFI 332 inhibits *Escherichia coli* O157:H7 biofilm formation. Appl. Microbiol. Biotechnol. 96, 1071–1078. 10.1007/s00253-012-3881-y 22274708

[B28] Lee J. Bansal T. Jayaraman A. Bentley W. E. Wood T. K. (2007). Enterohemorrhagic *Escherichia coli* biofilms are inhibited by 7-hydroxyindole and stimulated by isatin. Appl. Environ. Microbiol. 73, 4100–4109. 10.1128/AEM.00360-07 17483266 PMC1932762

[B29] Lejars M. Margaillan A. Bressy C. (2012). Fouling release coatings: a nontoxic alternative to biocidal antifouling coatings. Chem. Rev. 112, 4347–4390. 10.1021/cr200350v 22578131

[B30] Li C. Zhou L. Yang H. Lv R. Tian P. Li X.. (2017). Self-assembled exopolysaccharide nanoparticles for bioremediation and green synthesis of noble metal nanoparticles. ACS Appl. Mater. Interfaces. 9, 22808–22818, 10.1021/acsami.7b02908 28613815

[B31] Li H. T. Kang Z. H. Liu Y. Lee S. T. (2012). Carbon nanodots: synthesis, properties and applications. J. Mater. Chem. 22, 24230–24253. 10.1039/c2jm34690g

[B32] Li S. Yan X. Yang Z. Yang Y. Liu X. Zou J. (2014). Preparation and antibacterial property of silver decorated carbon microspheres. Appl. Surf. Sci. 292, 480–487. 10.1016/j.apsusc.2013.11.166

[B33] Lin F. Li C. Dong L. Fu D. Chen Z. (2017). Imaging biofilm-encased microorganisms using carbon dots derived from L. plantarum. Nanoscale. 9, 9056–9064. 10.1039/C7NR01975K 28639672

[B34] Liu Y. Busscher H. J. Zhao B. Li Y. Zhang Z. van der Mei H. C.. (2016). Surface-adaptive, antimicrobially loaded, micellar nanocarriers with enhanced penetration and killing efficiency in Staphylococcal biofilms. ACS Nano 10, 4779–4789. 10.1021/acsnano.6b01370 26998731

[B35] Nandi S. Malishev R. Parambath Kootery K. Mirsky Y. Kolusheva S. Jelinek R. (2014). Membrane analysis with amphiphilic carbon dots. Chem. Commun. 50, 10299–10302. 10.1039/C4CC03504F 25057851

[B36] Nandi S. Ritenberg M. Jelinek R. (2015). Bacterial detection with amphiphilic carbon dots. Analyst 140, 4232–4237. 10.1039/C5AN00471C 25919018

[B37] Natalio F. Andre R. Hartog A. F. Stoll B. Jochum K. P. Wever R.. (2012). Vanadium pentoxide nanoparticles mimic vanadium haloperoxidases and thwart biofilm formation. Nat. Nanotechnol. 7, 530–535. 10.1038/nnano.2012.91 22751222

[B38] Nguyen T. K. Duong H. T. Selvanayagam R. Boyer C. Barraud N. (2015). Iron oxide nanoparticle-mediated hyperthermia stimulates dispersal in bacterial biofilms and enhances antibiotic efficacy. Sci. Rep. 5:18385. 10.1038/srep18385 26681339 PMC4683393

[B39] Nguyen T. K. Selvanayagam R. Ho K. K. K. Chen R. Kutty S. K. Rice S. A.. (2016). Co-delivery of nitric oxide and antibiotic using polymeric nanoparticles. Chem. Sci. 7, 1016–1027. 10.1039/C5SC02769A 28808526 PMC5531038

[B40] Shivapooja P. Yu Q. Orihuela B. Mays R. Rittschof D. Genzer J.. (2015). Modification of silicone elastomer surfaces with zwitterionic polymers: short-term fouling resistance and triggered biofouling release. ACS Appl. Mater. Interfaces 7, 25586. 10.1021/acsami.5b09199 26554418

[B41] Reckmeier C. J. Schneider J. Susha A. S. Rogach A. L. (2016). Luminescent colloidal carbon dots: optical properties and effects of doping [Invited]. Opt. Express 24, A312–A340. 10.1364/OE.24.00A312 26832584

[B42] Ren D. Sims J. J. Wood T. K. (2001). Inhibition of biofilm formation and swarming of *Escherichia coli* by (5Z)-4-bromo-5-(bromomethylene)-3-butyl-2(5H)-furanone. Environ. Microbiol. 3, 731–736. 10.1046/j.1462-2920.2001.00249.x 11846763

[B43] Ren D. Zuo R. Gonzalez Barrios A. F. Bedzyk L. A. Eldridge G. R. Pasmore M. E.. (2005). Differential gene expression for investigation of *Escherichia coli* biofilm inhibition by plant extract ursolic acid. Appl. Environ. Microbiol. 71, 4022–4034. 10.1128/AEM.71.7.4022-4034.2005 16000817 PMC1169008

[B44] Ritenberg M. Nandi S. Kolusheva S. Dandela R. Meijler M. M. Jelinek R. (2016). Imaging *Pseudomonas aeruginosa* biofilm extracellular polymer scaffolds with amphiphilic carbon dots. ACS Chem. Biol. 11, 1265–1270. 10.1021/acschembio.5b01000 26882175

[B45] Robinson A. M. Bannister M. Creeth J. E. Jones M. N. (2001). The interaction of phospholipid liposomes with mixed bacterial biofilms and their use in the delivery of bactericide. Colloids Surf. A 186, 43–53. 10.1016/S0927-7757(01)00481-2

[B46] Taglietti A. Arciola C. R. D'agostino A. Dacarro G. Montanaro L. Campoccia D.. (2014). Antibiofilm activity of a monolayer of silver nanoparticles anchored to an amino-silanized glass surface. Biomaterials 35, 1779–1788. 10.1016/j.biomaterials.2013.11.047 24315574

[B47] Wang L. Zhou H. S. (2014). Green synthesis of luminescent nitrogen-doped carbon dots from milk and its imaging application. Anal. Chem. 86, 8902–8905. 10.1021/ac502646x 25181643

[B48] Wang Y. Hu A. (2014). Carbon quantum dots: synthesis, properties and applications. J. Mater. Chem. C 2, 6921–6939. 10.1039/C4TC00988F

[B49] Wood T. K. (2009). Insights on Escherichia coli biofilm formation and inhibition from whole-transcriptome profiling. Environ. Microbiol. 11, 1–15. 10.1111/j.1462-2920.2008.01768.x 19125816 PMC2621073

[B50] Yang J. Zhang X. Ma Y. H. Gao G. Chen X. Jia H. R.. (2016). Carbon dot-based platform for simultaneous bacterial distinguishment and antibacterial applications. ACS Appl. Mater. Interfaces 8, 32170–32181. 10.1021/acsami.6b10398 27786440

[B51] Yang L. Jiang W. Qiu L. Jiang X. Zuo D. Wang D.. (2015). One pot synthesis of highly luminescent polyethylene glycol anchored carbon dots functionalized with a nuclear localization signal peptide for cell nucleus imaging. Nanoscale 7, 6104–6113. 10.1039/C5NR01080B 25773263

[B52] Yang X. Luo Y. Zhu S. Feng Y. Zhuo Y. Dou Y. (2014). One-pot synthesis of high fluorescent carbon nanoparticles and their applications as probes for detection of tetracyclines. Biosens. Bioelectron. 56, 6–11. 10.1016/j.bios.2013.12.064 24445067

[B53] Zhang R. Chen W. (2014). Nitrogen-doped carbon quantum dots: facile synthesis and application as a “turn-off” fluorescent probe for detection of Hg^2+^ ions. Biosens. Bioelectron. 55, 83–90. 10.1016/j.bios.2013.11.074 24365697

[B54] Zhang Y. Shen Y. Teng X. Yan M. Bi H. Morais P. C. (2015). Mitochondria-targeting nanoplatform with fluorescent carbon dots for long time imaging and magnetic field-enhanced cellular uptake. ACS Appl. Mater. Interfaces 7, 10201–10212. 10.1021/acsami.5b00405 25942702

[B55] Zhao H. X. Liu L. Q. Liu Z. D. Wang Y. Zhao X. J. Huang C. Z. (2011). Highly selective detection of phosphate in very complicated matrixes with an off-on fluorescent probe of europium-adjusted carbon dots. Chem. Comm. 47, 2604–2606. 10.1039/c0cc04399k 21234476

